# Investigating the Buffering Effects of Greenery on the Adverse Emotional, Mental and Behavioral Health during the Pandemic Period

**DOI:** 10.3390/ijerph19148749

**Published:** 2022-07-18

**Authors:** Paolo Contini, Santo Di Nuovo, Maria Sinatra, Elisabeta Osmanaj, Lucia Monacis

**Affiliations:** 1 Inter-University Centre of Research in Population, Environmental and Health (CIRPAS), University of Bari, 70121 Bari, Italy; paolo.contini@uniba.it; 2 Department of Education, University of Catania, 95124 Catania, Italy; sdinuovo@unict.it; 3 Institute of Design, 75100 Matera, Italy; maria.sinatra@ssmlnelsonmandela.it; 4 Faculty of Education Sciences, University of Elbasan “Aleksandër Xhuvani”, 3001 Elbasan, Albania; eli.osmanaj@gmail.com; 5 Department of Humanities, University of Foggia, 71121 Foggia, Italy

**Keywords:** COVID-19, greenery, buffering effects, emotional health, mental health, behavioral health

## Abstract

In light of the adverse emotional, mental and behavioral outcomes caused by the pandemic period, this research analyzed the associations between emotional distress and poor health outcomes and the buffering effects of greenery on these outcomes. An online cross-sectional survey between June–November 2021 was distributed among 1314 young Italian adults. Bivariate associations and multivariate regression analyses were applied to the data. Findings showed that emotional distress was positively related to poor mental health outcomes and to some of the unhealthy behaviors. In addition, green pathways differently impacted on health: the indoor features confirmed buffering effects on adverse emotional and mental health responses, whereas the outdoor features played no salutogenic role. In conclusion, whereas the outbreak period of the pandemic has led to the rediscovering/reinforcement of the attachment to nature to cope with negative affective states, the successive waves characterized by selected limitations and new living rules of social adaptation may have brought about a reduced affinity toward nature. Target interventions in terms of biophilic design for indoor environmental sustainability are needed in order to increase the innate human–nature connection and thus to promote public health.

## 1. Introduction

During the COVID-19 sanitary emergency, there have been contradictory effects of safety measures on mental health. Although some of the negative affective states have been declined (i.e., loneliness and apathy) or remained stable (i.e., boredom) during the early outbreak, divergent trends have been observed as the lockdown has continued: frustration has risen, whereas loneliness and sadness, after sharply increasing, have remained stable [1]. Loneliness and social isolation have showed relatively little overall change before and during the pandemic. This has led researchers to align with the idea of substitution [2], which refers to individuals’ ability to find new creative ways of social connection [3] to fulfil their fundamental need to belong in the face of the blocking of family and social ties. Increased feelings of loneliness and sadness may have led to the rediscovery of an attachment to the natural world or to the development of connectedness to it.

By linking the biophilia hypothesis [4] to the psychological framework of the need to belong [5], connectedness to nature may satisfy this need. Supporting Ulrich’s stress reduction theory (SRT) [6] and Kaplan’s attention restorative theory (ART) [7], prior studies have generally provided empirical evidence for the restorative effect of exposure to nature on some aspects of psychological health, such as attentional resources [8] or mental health [9]. In addition, various (i.e., intentional, direct, indirect or incidental) forms of nature exposure have been generally considered as a potential salutogenic factor of enhancing resilience that positively impacts on affective and cognitive functioning of individuals [10]. Investigations carried out during the first year of the pandemic have confirmed positive effects of nature exposure on psychological health [11,12].

However, after the phase of the new normality corresponding to the period June–September 2020, other waves occurred in Italy, leading to the three-tiered system of restrictions. The three tiers were labelled according to a color scheme: yellow, orange and red, each of them corresponded to the increasing levels of restrictions. The tiers were assigned to each of the regions and areas after an epidemiological risk assessment by the Italian Ministry of Health; they were based on the combination of different quantitative indicators, such as the level of transmission, the burden on older age groups and healthcare and resilience of monitoring systems. Although the three-tiered system of restrictions caused a lower impact on human activities and a large reduction in daily hospitalization [13], negative effects on psychological health outcomes have been reported once again: increased emotional discomfort, feelings of worthlessness, hopelessness, helplessness and inability to see an end to the pandemic [14], insomnia [15] and worsened sleep quality that was significantly associated with increased negative dream emotionality [16]. Other unhealthy behaviors have also been identified: increase in Internet and social media use, abuse of alcohol, tobacco and drugs [17,18] and increase in online/offline gaming behavior and eating disorders [19,20].

As individuals have become accustomed to fluctuating negative emotional states as a result of the pandemic conditions, it has also been reasonable to encounter a sort of durable resilience based on an innate or developed ecological approach encapsulating the nature connectedness that could help individuals stay physically and mentally healthy during ongoing social distancing. On this premise, the current study focused on the individuals’ contact with nature that enhances psychological restoration, taking into account different scenarios of engagement with nature, such as indoor vegetation, green window views and green access to both private and public green spaces.

Much research has examined the potential role of nature exposure in mitigating the worsening effects on emotional, mental and behavioral health during the pandemic period [21,22,23,24,25,26,27]. However, limited studies were conducted in the Italian context. Consequently, the current research aimed at exploring (i) the associations between increased levels of emotional distress and poor health outcomes; (ii) the buffering effects of the contact with nature on the adverse emotional, mental and behavioral health.

To these aims, the following hypotheses will be tested: higher levels of negative emotions are associated with poor levels of mental and behavioral health outcomes (H1), effects of greenery features reduce negative affective states (H2_1_), cognitive functioning impairment (H2_2_) and poor level of unhealthy behaviors (H2_3_). The last hypothesis is formulated following Corley and colleagues’ work [23], which indicates significant effects of nature exposure—in terms of private and public green access—on healthy behavioral outcomes.

## 2. Materials and Methods

### 2.1. Survey Design

A cross-sectional survey between June–November 2021 was used to measure (i) negative affective states and poor mental and behavioral outcomes as the main dependent variables; (ii) the effects of the two types of nature exposure, i.e., green views and green access, in mitigating the negative outcomes. Due to a lack of well-validated indicators of interest, in line with some studies [12,21] this survey was an ad hoc self-reported questionnaire designed by the authors. Using a snowball sampling technique, the questionnaire was distributed to a sample of 1379 Italian adults via an online survey platform using Google Forms. The link to the survey was published on selected social media (e.g., WhatsApp, Facebook and Instagram). A priori sample size was also calculated to make inferences about the population based on our sample. The minimum required sample size was 136 participants with effect size F^2^ = 0.15 α = 0.05, 1 − β = 0.90 and nine predictors. Due to the issues of reliability of data from online surveys, data from participants with missing or incomplete responses (around 5%; *n* = 65) were removed a priori from the dataset. Consequently, the final estimation sample was 1314 and was above the minimum required for all analyses.

The study was conducted in accordance with the ethical standards of the Ethical Code of the Italian Association of Psychology (AIP) and with the European Code of Conduct for Research Integrity (ECCRI) on human experimentation, complied with the Declaration of Helsinki. The survey protocol was also approved by the local Ethics Commission of the University of Bari (code number: P051/022021). The anonymity of the results was guaranteed as no personal information was gathered from the participants. All participants signed an online informed consent, and they could withdraw from the survey at any time and for any reason.

### 2.2. Measures

#### 2.2.1. Sociodemographic Variables

The first section included information on participants’ sociodemographic characteristics, i.e., age, gender, level of education (middle school, high school and degree) and occupation (student, worker, unemployed and unoccupied) and the working mode during the second and third waves of COVID-19 (November 2020–April 2021) (e.g., smart or non-smart working).

#### 2.2.2. Emotional States

The second section was divided into two parts. The first part dealt with the main emotional states experienced during the pandemic period (November 2020–April 2021). Participants self-assessed their emotions from a list of 28 negative emotional states underlying the main basic negative affective states (anger, disgust, fear, sadness and surprise) on the basis of the hybrid model underlying Plutchik’s wheel of emotions [28].

The second part assessed the intensity level of each negative emotional state by comparing the level of intensity experienced in the period November 2020–April 2021 with the level of intensity experienced in the period of the new normality (June–September 2020). Participants indicated to what extent they experienced an increasing of intensity levels for each affective state underlying the main negative emotion (e.g., “In comparison to the period of June–September 2020, indicate if you agree or disagree with the following actual affective state”, “I feel more vulnerable than before”, and “I feel lonelier than before“) by using a 5-point Likert scale (from 1 Strongly disagree to 5 Completely agree). Low scores correspond to the first level indicating no change, mean score to the second level indicating neutral and high score to the last level, namely high levels of intensity (measured as an ordinal variable). A total score was also calculated with higher values indicating a marked worsening in each emotional cluster.

#### 2.2.3. Health Outcomes

This part included a short questionnaire composed of 10 items assessing the increase in mental and behavioral unhealthy lifestyles during November 2020–April 2021. Each participant was asked to express his/her agreement with an increase in the levels of poor health outcomes, such as sleep disturbance, recurrent thoughts and dreams, poor concentration, eating disorders, online behaviors (the amount of time spent on online gaming and internet and social networks) and drug, tobacco and alcohol consumption. A 5-point Likert scale from 1 Strongly disagree to 5 Completely agree was applied. Disagreement indicated no change in the health state; a low score no change and a high score a marked worsening in the health outcome.

#### 2.2.4. Nature Experience: Contact with Nature

The third section of the survey included three items regarding indoor and outdoor greenery features. The first dealt with indoor vegetation, i.e., the presence of potted plants (yes/no). The second was related to the indirect contact with nature, the amount of green visible from the windows of the home. It was assessed using a 5-point Likert scale from zero to the highest amount of green view (“no green space” = 1, “a limited view of green” with outdoor plants and shrubs but without trees = 2, “a view of a small amount of green” including a limited number of trees (from one to three trees), outdoor plants and shrubs = 3, “a view of a large amount of green” including a broader number of trees, outdoor plants and shrubs = 4 and “a view entirely of green features” including trees, plants, flowers/gardens or parks = 5). The third item referred to outdoor green features or direct contact with nature, i.e., accessibility to private green spaces (terrace or courtyard with greenery or private garden) and to public/urban green environments (parks, countryside or mountains) during the COVID-19 pandemic. The amount of direct contact was assessed using a categorical variable identifying three different categories: no access = 1, private access = 2 and public access = 3.

### 2.3. Analysis Strategy

Preliminary, descriptive statistics were performed to examine participants’ characteristics. At the inception of the inferential statistical techniques, the reliabilities of the measures, such as values of Cronbach’s alpha and McDonald’s omega were examined, and the normal distribution of the data was checked using values of skewness, kurtosis and z-scores for the emotional intensity levels and for each health outcome. The associations of the intensity level of the five negative emotions (measured as ordinal) with the poor health outcomes (measured as continuous) were examined using Spearman correlation coefficients. Ordinal logistic regression (OLR) for the levels of each emotional state and ordinary least squared regression (OLS) with multicategorical variables for each health outcome were performed, in order to unpack the effects of indoor/outdoor greenery features on the emotional intensity level and health outcomes. All the models were adjusted with sociodemographic variables, such as age, gender (coded as male = 1 and female = 2), occupation (as ordinal with unoccupied as the reference category), working mode (coded as smart working condition = 1 and non-smart working condition = 2) and education level (as ordinal with degree as the reference category). Greenery features indicators referred to the amount of green view (as a continuous variable), plants at home (coded as plants at home = 1 and no plants at home = 2) and the type of green access (as ordinal variable with public access as the reference category). The main assumptions were checked: for OLR, the assumption of the proportional odds (PO), i.e., that the parameters were the same across all categories, was checked by running the test of parallel lines. A *p*-value greater than 0.50 was the indicator to be met. Where the test of parallel lines violated this assumption, the multinomial logistic regression (MLR) was used. The assumption of no multicollinearity of the independent variables was also tested using Spearman correlation analysis and the variance inflation factor (VIF). Variables with values higher than 0.70 for bivariate associations and 3.00 for VIF indicated multicollinearity issues and were omitted from the subsequent analyses. The variable age was centered in both OLR and OLS analyses.

## 3. Results

### 3.1. Sample Characteristics

Descriptive statistics were run to analyze participants’ sociodemographic information and the distribution of greenery (Table 1). The average age was 33.52 years, ranging from 18 to 72 in the total sample; the female gender was the most prevalent (almost 80%) and the degree was the most representative level of education (54%). Slightly more than half of the respondents were workers (52%) in a non-smart working mode (55%). The degree was the highest level of education cited by both sexes (52% for males and 54% for females). As far as working status was concerned, more than half of the males worked (65%), whereas females were almost equally distributed between the student (48%) and worker (4%) categories. Finally, just over half of the respondents for both sexes reported working in non-smart mode during the pandemic restrictions. Information about nature features, such as indoor plants, and the data distribution concerning direct and indirect contact with nature are reported in Table 1. Generally, around one third of the respondents reported a view of a large amount of green from the windows of their home, and most of them had plants at home and private access to greenery features. Data on mean scores, standard deviation, kurtosis, skewness and z-scores for the level of intensity of each basic emotions and for health outcomes reported in the total sample are displayed in Table 2. Since the absolute values of skewness and kurtosis less than the recommended value of 1.00, and all the absolute z-scores of skewness and kurtosis are below the absolute value of 1.96, the normality assumptions were met, and thus parametric statistics were applied to the data.

### 3.2. Associations of Levels in Emotional Distress with Poor Health Outcomes

Results from correlation analysis showed no significant associations of drug consumption and time spent on gaming with all negative emotional states, as well as no association of the increase in internet and social media use with disgust. However, time spent in internet and social media was positively associated with the remaining negative emotions. Positive associations were generally observed between the other behavioral variables and negative affective states: unhealthy eating behaviors with high intensity levels in sadness, anger and surprise; abuse of tobacco with high levels of sadness; abuse of alcohol with high intensity levels of sadness and anger. Poor mental health outcomes (sleep disturbance, recurrent dreams and thoughts and poor concentration) were linked to all negative emotions (Table 3).

### 3.3. The Effects of Greenery Features on Levels of Emotional Distress and Unhealthy Outcomes

Regression analyses were carried out to investigate the effects of greenery features on the levels of each emotional state and on unhealthy outcomes. OLR analyses were performed for sadness, disgust, anger and fear, since none of the independent variables showed multicollinearity and all the values were less than 2.50, below the recommended threshold levels. As regards surprise, although data from model fit information showed an improvement in the model and a significant difference in the unexplained variation between the null and the final model, χ^2^(12) = 31,455, *p* = 0.002, results from the test of parallel lines indicated that the assumption PO was violated, χ^2^(12) = 26,419, *p* = 0.009. Consequently, MLR analysis was run to explore the relationships between greenery features and the level of surprise. All results are reported in Table A1 and Table A2 in Appendix A.

### 3.4. Sadness

Gender and level of education were positive predictors, whereas working conditions and views of green were negative predictors. Specifically, individuals identifying as female and with lower level of education were at higher risk of experiencing higher levels of sadness. Moreover, the likelihood of being at the highest levels of sadness was lower in non-smart working condition. Finally, as regards green viewing, data showed a decreasing probability of being in a higher level of this affective state when scores for views of greenery increased.

### 3.5. Disgust

Working conditions and viewing greenery were negative predictors. Individuals identifying in non-smart working mode and with greater views of greenery were at lower risk of experiencing a high level of disgust. The odds ratio of less than one indicated a decreasing probability of being in a higher level of this affective state when scores increased on the aforementioned variables.

### 3.6. Anger

Age and views of greenery were negative predictors, whereas educational level was a positive predictor. People with a middling level of education were more likely to fall into the higher level of anger than were those with a degree. As regards views of greenery and age, data showed a decreasing probability of being in a higher level on this emotional state when scores increased on the amount of visible green and age.

### 3.7. Fear

Gender and views of green were positive and negative predictors, respectively: individuals identifying as female were at higher risk of experiencing higher levels of worsening. In addition, a decreasing probability of being in a higher level of emotional state of fear was associated to a greater view of green.

### 3.8. Surprise

Results from MLR analysis indicated that the full model showed a significant improvement in fit over a null model and that the model fit the data well, since both deviance and Pearson chi-square tests were not significant (Table A2 in Appendix A). The first set of coefficients represents comparisons between no change of intensity level (baseline/reference group) and neutral (as comparison). The availability of views of greenery was a negative predictor: individuals scoring higher on this variable were less likely to experience a neutral level in the intensity, whereas the likelihood of falling into the “no-change” category was greater. The second set of coefficients showed a comparison between the no-change in intensity level (baseline/reference group) and a high level of emotional intensity. Only availability of views of greenery and working mode were negative predictors: individuals with high scores on views of greenery and who were in non-smart working mode were less likely to experience an emotional worsening, whereas the likelihood of falling into the “no-change” category was greater.

### 3.9. Health Outcomes

Results from single-exposure regression models indicated that the presence of plants at home was significantly associated with a lower self-reported increase in sleep disturbance and poor concentration, whereas the amount of greenery viewed from home windows was significantly associated with a lower self-reported increase in sleep disturbance, recurrent dreams and thoughts and poor concentration. Among socio-demographic factors, being female was positively associated with a higher self-reported increase in sleep disturbance, recurrent dreams and thoughts and being young was associated with a marked worsening in recurrent dreams, thoughts and poor concentration. Finally, being a worker was associated with a lower increase in unhealthy behaviors connected to sleep disturbance in comparison to being unoccupied. No significant associations were found between views of greenery and the remaining poor health outcomes (abuse of tobacco, drug and alcohol, eating disorders, time spent on internet and on online/offline gaming), nor did any associations emerge between different types of green access and all unhealthy outcomes. Only the significant associations between poor health outcomes and green-related variables were reported in Table 4.

## 4. Discussion

In light of the mixed effects of lockdown restrictions on emotional, mental and physical health, the current study examined (i) the associations between emotional distress and poor health outcomes and (ii) the buffering effects of nature exposure in reducing adverse emotional, mental health and behavioral outcomes.

Results from correlations generally confirmed the supposed relationships (H1), showing positive associations of emotional distress with poor health outcomes, and supporting evidence of how, beyond the well-documented symptoms of anxiety and depression [29], emotional repercussions affected mental health vulnerability and unhealthy lifestyles.

Among the unhealthy behaviors, the observed positive associations between eating dysfunction/adverse changes in eating behavior and negative emotions were in line with the investigations carried out in the pandemic period [19,20], and with the common assumption that the experience of negative emotions can lead to overeating, the so-called “emotional eating” [30]. Indeed, when trying to deal with the negative experience of self-isolation and the negative emotions it causes, like sadness, anger and surprise, people seemed to be at higher risk of overeating, (perhaps) as a result of being more prone not only to look for reward and gratification but also to consider food intake as a way to escape monotony. This finding was in contrast with the study by Caso and colleagues [31] reporting no association between negative emotions and unhealthy food consumption during the pandemic period. Maybe this inconsistency was due to the instrument (short version) used to assess only five negative affective states. Consequently, further studies should be carried out to better clarify the finding.

As for the increased use of internet and social media, the data indicated no association with disgust but positive associations with the remaining negative emotions. During the pandemic crisis, internet usage and social media might alleviate the adverse emotional impact of isolation by releasing tension and through emotional catharsis [32].

With regard to the other unhealthy behavioral outcomes, higher levels of sadness were significantly linked to an increase in both tobacco and alcohol consumption, whereas higher levels of anger were associated with increased alcohol consumption. As sadness-related emotions (e.g., depression, loneliness and despair) are oriented to a compensatory tendency to reward-seeking behaviors, they might elicit an implicit motivational drive to re-establish equilibrium and replace loss through enhanced substitution, thus leading to a rise in smoking and alcohol intake. Such findings were in line with investigations generally reporting increases in rates of tobacco and alcohol consumption during the pandemic [17,18], and in contrast with Izzo and colleagues’ study [33], which reported a decrease in alcohol consumption, although these data related to the period April–May 2020. Furthermore, the current findings provided empirical evidence for a significant connection between sadness and addictive substance use, as reported before the pandemic [34]. As individuals became more frustrated with, critical of and hostile to others who did not comply with safety rules, the significant association of anger with the abuse of alcohol may have been the result of a tendency to abuse of alcohol as a source of relief to soothe angry feelings, but often with the opposite effect. No significant associations were found between negative emotions and either increased drug consumption or overuse of online gaming, suggesting that individuals were not at risk of developing such problematic/addictive behaviors before the COVID-19 restrictions. In this sense, results may indicate that: 1. unlike individuals affected by substance use disorders and problematic gaming behaviors [17], the general population does not use drugs and gaming as coping strategies to reduce the adverse feelings of prolonged isolation and loneliness, and 2. the use of rewarded behaviors/actions (e.g., online gaming) as a dysfunctional coping strategy could exacerbate pre-existing addictive behaviors. Nevertheless, longitudinal studies on drug and game abuse before and during the COVID-19 pandemic are needed to throw light on this controversial issue.

As regards unhealthy mental outcomes, results yielded evidence of positive associations of emotional distress with poor mental health outcomes (sleep disturbance, recurrent thoughts and dreams and poor concentration). They corroborated prior data demonstrating that, such as individuals with pre-existing mental disorders, those who showed higher levels of negative emotional health turned out to be at high risk of poor mental health outcomes. Indeed, the general population, patients and workers engaged in public health experienced a variety of negative emotions, such as anxiety over uncertain outcomes, guilt, perception of impotence, loneliness, frustration, worthlessness and lack of hope, which activate cognitive mechanisms including repetitive health worries, ruminations and catastrophic thinking [14]. Similarly, stress, negative emotions and repetitive negative thinking were also associated with problems in initiating and maintaining sleep [15] and increased negative dream emotionality [16].

Regarding the buffering effects of greenery features on emotional, mental and behavioral health outcomes, findings partially confirmed the salutogenic effect of nature experience on health responses. Indeed, consistently with H2_1_ on the restorative affective effects of nature exposure, results indicated that viewing greenery from the windows reduced the risk of higher level of negative emotions. As for H2_2_ on the cognitive functioning effects of nature exposure, as expected, findings showed that viewing greenery and having plants at home had positive effects in reducing poor mental health outcomes. The buffering effect of viewing nature as a form of indirect contact with nature, that was found to be the common features to emotional and cognitive health, was in line with Soga et al.’s assumption [34] that the “less” immediate experience with nature was likely to promote lower levels of emotional and mental distress. In fact, findings indicated that this experience brings more benefits to human mind than the other scenarios (indoor vegetation and outdoor features), probably because of the multisensorial features underlying viewing nature. Moreover, the presence of indoor vegetation was found to have beneficial effects on cognitive functioning, such as sleep disturbance and poor concentration, thus extending and corroborating previous data regarding mental health benefits in living environments [8,9,35] and affective states of COVID-19 home confinement [21,22,24].

Surprisingly, in contrast with H2_3_ and inconsistently with prior literature [21,22,23,24,25,26,27], findings showed no beneficial effects of direct contact on emotional, mental and behavioral health, implying that public and private greenspace played no meaningful role in lessening adverse health. As suggested [34], levels of emotional and mental health can be improved without physically or intentionally visiting natural environments. The unexpected absence of significant effects of outdoor green features could be linked to the concept of the extinction of nature experience [34]: because of the limited opportunities for people living in urban areas to interact with nature and the rise of virtual alternatives for leisure-time activities (social media and internet) [36], the progressive loss of human–nature interactions might accelerate the loss of memory of the (direct) non-use value of nature. The observed lack of significant effects was in line with prior research showing that individuals, who do not directly interact with nature, were less likely to perceive and value the advantages of such interactions [37], were likely to lose short-term and long-lasting benefits associated with health and wellbeing [38] and were less motivated to want to visit and protect nature [39].

This investigation is one of the first studies that provides a picture of how different greenery features might have positively affected emotional, mental and behavioral health. Despite this strength, certain limitations should be acknowledged. First, the research design cannot definitively establish a cause-effect relationship. Second, self-reported data may lead to bias. Next, data were collected via an online-based questionnaire that may have limited to specific social groups. In addition, the measurement of the access to greenspace (direct contact) based on a single item and without other information such as the frequency or duration of the contact and other related activities (gardening, green-exercise, etc.) may have given a non-exhaustive overview of human–nature connectedness. Future investigations should take account of these limitations to better explore these issues.

## 5. Conclusions

Taken as a whole, the study showed that nature experience differently impacted on health during the pandemic period: although the buffering effects of indirect contact on adverse emotional and mental health responses were confirmed, direct contact was unlikely to play a significant salutogenic role, probably due to reduced connectedness with nature. Indeed, whereas the outbreak period with full social restrictions led to the rediscovering/reinforcement of the attachment to the natural world as a source of resilience to cope with negative affective states, the following waves of the COVID-19 pandemic, being characterized by partial and selected limitations and new living rules of social adaptation, may have brought about a reduced affinity toward nature. This suggests that there are two sides of the human–nature relationship: a state/fluctuating human–nature connection for direct contact and a more durable/authentic connection for indirect contact. In sum, the current study calls a more intentional urban planning and architectural approach to housing and the implementation of government policies toward the promotion of green spaces, not just as “beautiful” components of the cities but also as constitutive elements of the human building environment for the health of its inhabitants.

## Figures and Tables

**Table 1:** Sociodemographic characteristics and data distribution for indoor and outdoor greenery features.

	*N* = 1314
*Age years*	33.52 ± 12.162
*Gender M/F*	264/1050
*Educational level*	
Middle school	26
High school	578
Degree	710
*Job*	
Student	599
Worker	670
Unemployed	19
Unoccupied	26
*Working condition*	
Smart worker	605
No-smart worker	709
*Indoor nature features*	
Plants at hone Yes/No	920; 394
*Green vision*	
No vision	104
Limited vision	267
Little vision	338
Much vision	465
Full vision	140
*Outdoor features*	
No access	187
Private access	788
Public access	339

**Table 2:** Mean scores, standard deviation, values of kurtosis, skewness and their relative standard errors for the mean score of each emotion and for each item of the health outcome.

	Mean (SD)	Kurtosis (SE)	Skewness (SE)
*Emotion*			
Sadness	2.94 (0.888)	−0.366 (0.274)	−0.178 (0.138)
Disgust	3.11 (0.899)	−0.284 (0.274)	−0.487 (0.138)
Anger	2.96 (0.780)	0.074 (0.274)	−0.172 (0.138)
Fear	3.11 (0.903)	−0.414 (0.274)	−0.405 (0.138)
Surprise	3.12 (0.885)	−0.007 (0.274)	−0.517 (0.138)
Cronbach’s α 0.887			
McDonald’s ω 0.889			
*Health*			
Sleep disturbance	3.16 (1.244)	−1.015 (0.274)	0.062 (0.138)
Recurrent thoughts	2.76 (1.112)	−0.71 (0.274)	−0.867 (0.138)
Recurrent dreams	3.57 (1.146)	−0.079 (0.274)	−0.368 (0.138)
Poor concentration,	3.32 (1.193)	−0.933 (0.274)	0.755 (0.138)
Eating disorders	2.19 (1.382)	−0.808 (0.274)	0.823 (0.138)
Drug consumption	1.94 (1.086)	−0.40 (0.274)	1.883 (0.138)
Tobacco consumption	1.49 (0.862)	3.269 (0.274)	0.057 (0.138)
Alcool consumption	2.72 (1.258)	−1.172 (0.274)	−0.987 (0.138)
Time spent on Internet and social media	3.84 (1.063)	0.577(0.274)	0.778 (0.138)
Time spent on online gaming	2.08 (1.157)	−0.438(0.274)	0.062 (0.138)
Cronbach’s α 0.792 McDonald’s ω 0. 779			

**Table 3:** Association between change in intensity levels of each emotional cluster and unhealthy outcomes.

	Sadness	Disgust	Angry	Fear	Surprise
Sleep disturbance	**0.437** **	**0.303** **	**0.364** **	**0.446** **	**0.317** **
Recurrent dreams	**0.347** **	**0.217** **	**0.340** **	**0.303** **	**0.229** **
Recurrent thoughts	**0.436** **	**0.315** **	**0.396** **	**0.472** **	**0.380** **
Poor concentration	**0.429** **	**0.317** **	**0.421** **	**0.429** **	**0.380** **
Abuse of tobacco	**0.139** **	−0.022	0.100	0.042	0.051
Abuse of alcohol	**0.137** *	−0.004	**0.118** **	0.016	0.087
Abuse of drug	0.107	0.039	0.072	0.012	−0.002
Eating disorders	**0.241** **	0.078	**0.211** **	0.074	**0.127** *
Internet and social media	**0.214** **	0.078	**0.255** **	**0.240** **	**0.113** *
Gaming	0.050	−0.044	0.096	0.060	0.059

Significant values are in bold; * *p* < 0.050; ** *p* < 0.010.

**Table 4:** Results of single-exposure regression models of the associations between nature exposure and the significant health outcomes.

	Sleep Disturbance	Recurrent Dreams	Recurrent Thoughts	Poor Concentration
	R^2^ = 0.153, AR^2^ = 0.119, F = 4.539 ***	R^2^ = 0.128, AR^2^ = 0.093, F = 3.680 ***	R^2^ = 0.210, AR^2^ = 0.179, F = 60.683 ***	R^2^ = 0.143, AR^2^ = 0.109, F = 4.177 ***
	β [95% CI]	β [95% CI]	β [95% CI]	β [95% CI]
*Gender*	**0.241** [0.415 10.076] ***	**0.154** [0.127 0.726] **	**0.153** [0.142 0.730] **	0.083 [−0.074 0.565]
*Age centered*	−0.128 [−0.028 0.002]	**−0.186** [−0.031 −0.003] *	**−0.258** [−0.038 −0.011] ***	**−0.193** [−0.034 −0.004] *
Education level = midldle school	0.035 [−0.677 1.266]	0.046 [−0.536 10.227]	0.058 [−0.413 1.315]	−0.035 [−1.221 0.655]
Education level = high school	0.012 [−0.237 0.299]	0.066 [−0.096 0.391]	0.038 [−0.151 0.326]	0.067 [−0.099 0.419]
Work = Student	−0.342 [−1.822 −0.112]	0.146 [−0.552 10.203]	−0.233 [−10.397 0.324]	−0.265 [−1.568 0.300]
Work = Worker	**−0.376** [−1.845 −0.024] *	0.085 [−0.637 10.015]	−0.337 [−10.581 0.039]	−0.310 [−1.618 0.141]
Work = Unemployed	−0.094 [−2.318 0.453]	0.000 [−1.255 1.260]	−0.077 [−10.936 0.529]	−0.010 [−1.436 1.239]
Smart/non-Smart Working	−0.029 [−0.338 0.196]	−0.053 [−0.361 0.124]	−0.058 [−0.370 0.105]	−0.087 [−0.466 0.050]
Plants at home	**−0.120** [−0.633 −0.030] **	−0.026 [−0.339 0.208]	−0.076 [−0.463 0.073]	**−0.116** [−0.600 −0.018] *
Green Views	**−0.225** [−0.378 −0.121] ***	**−0.182** [−0.296 −0.064] **	**−0.236** [−0.355 −0.127] ***	**−0.201** [−0.338 −0.090] **
No Green Access	−0.021 [−0.527–0.378]	−0.096 [−0.715 0.106]	−0.049 [−0.563 0.243]	0.048 [−0.274 0.599]
Private Green Access	0.058 [−0.166–0.462]	−0.007 [−0.300 0.270]	−0.006 [−0.295 0.265]	−0.009 [−0.326 0.281]

β = beta coefficient; R^2^ = R-squared; AR^2^ = adjusted R-squared; F = F statistic. * *p* < 0.05; ** *p* < 0.01; *** *p* < 0.001; significant beta values are in bold.

## Data Availability

The data that support the findings of this study are available on request from the corresponding author. The data are not publicly available due to privacy or ethical restrictions. Therefore, they are available from the corresponding author upon reasonable request.

## Appendix A

**Table A1:** Ordinal logistic models for the change in intensity level of each basic emotion.

	Sadness	Disgust	Anger	Fear
Model fit Information X^2^(df)	35,841(12) *******	34,835 (12) *******	35,150(12) *******	40,731(12) *******
Goodness of Fit				
Pearson Test X^2^(df)	595,183(564) ^ns^	604,963 (564) ^ns^	596,175(564) ^ns^	607,605 (564) ^ns^
Deviance Test X^2^(df)	618.783(564) ^ns^	608,153 (564) ^ns^	585.106(564) ^ns^	614,401 (564) ^ns^
Pseudo R^2^				
Cox e Snell/Nagelkerke/McFadden	0.108/0.122/0.053	0.105/0.119/0.052	0.106/0.122/0.055	0.122/0.138/0.060
Test of Parallel Lines	13,873(12) ^ns^	16,269 (12) ^ns^	12,547(12) ^ns^	9700(12) ^ns^
	OR [95% CI]	OR [95% CI]	OR [95% CI]	OR [95% CI]
*Gender*	**2.218** [1.292–3.808] ******	1.560 [0.909–2.685]	1.477 [0.846–2.580]	**2.574** [1.496–4.430] ******
*Age*	0.992 [0.968–1.017]	1.001 [0.986–1.016]	**0.962** [0.939–0.986] ******	0.986 [0.962–1.011]
Work = Student	0.588 [0.130–2.656]	0.408 [0.093–1.787]	0.626 [0.139–2.810]	0.648 [0.136–3.083]
Work = Worker	0.613 [0.150–2.503]	0.457 [0.113–1.856]	1.066 [0.260–4.365]	0.625 [0.140–2.783]
Work = Unemployed	0.727 [0.083–6.377]	0.371 [0.043–3.199]	1.204 [0.130–11.145]	0.525 [0.058–4.715]
Work = Unoccupied as reference				
SW NSW = Smart Working	**0.649** [0.422–1.00] *****	**0.530** [0.343–0.820] ******	0.649 [0.417–1.011]	0.771 [0.501–1.187]
Education level = Middle	**6.593** [1.089–39.898] *****	1.001 [0.978–1.026]	**5.773** [1.032–32.299] *****	4.143 [0.676–25.399]
Education level = High	1.467 [0.951–2.263]	2.500 [0.360–17.342]	0.906 [0.582–1.411]	1.005 [0.651–1.551]
Education level = Degree as reference				
Plants at home	0.849 [0.520–1.385]	0.550 [0.337–0.895]	0.628 [0.381–1.036]	0.682 [0.419–1.112]
Green views	**0.737** [0.597–0.910] ******	**0.711** [0.576–0.877] **	**0.760** [0.614–0.940] *****	**0.718** [0.581–0.888] **
Green Access = No access	1.017 [0.479–2.157]	0.726 [0.348–1.514]	1.695 [0.808–3.533]	1.534 [0.716–3.284]
Green Access = Private Access	0.802 [0.486–1.323]	0.642 [0.381–1.080]	0.870 [0.518–1.460]	0.845 [0.510–1.399]
Green Access = Pubic Access as reference				

OR < 1 indicates a decrease in the likelihood of showing change in intensity; OR > 1 equals to an increase in intensity; OR = Odds ratio; CI = Confident Interval; * *p* < 0.050; ** *p* < 0.010; *** *p* < 0.001; ns = Not significant; Significant values of OR are in bold.

**Table A2:** Multinomial logistic model for surprise.

	Surpise	
Model fit Information X^2^(df)	57,404(24) ***	
Goodness of Fit		
Pearson Test X^2^(df)	594,585 (552) ^ns^	
Deviance Test X^2^(df)	585,458 (552) ^ns^	
Pseudo R^2^		
Cox and Snell/Nagelkerke/McFadden	0.167/0.190/0.087	
**Baseline Group: No Change**	**Comparison Group** **Neutral**	**Comparison Group** **High Level of Intensity**
	**OR [95% CI]**	**OR [95% CI]**
*Gender*	0.842 [0.390–1.819]	1.014 [0.441–2.329]
*Age*	0.996 [0.962–1.031]	0.998 [0.961–1.035]
Work = Student	0.351 [0.028–4.334]	0.489 [0.039–6.176]
Work = Worker	0.406 [0.036–4.538]	0.403 [0.036–4.471]
Work = Unemployed	0.448 [0.046–4.410]	0.537 [0.064–5.981]
Work = Unoccupied as reference		
SW NSW = Smart Working	0.609 [0.318–1.163]	**0.328** [0.168 −0.644] ******
Education level = Middle	0.262 [0.014–4.951]	1.919 [0.168–2.863]
Education level = High	0.867 [0.479–1.679]	0.850 [0.440–1.641]
Education level = Degree as reference		
Plants at home	1.279 [0.631–2.591]	0.457 [0.206–1.011]
Green views	**0.616** [0.448–0.846] ******	**0.718** [0.516–1.000] *****
Green Access = No access	0.462 [0.145–1.477]	1.062 [0.342–3.293]
Green Access = Private Access	1.130 [0.529–2.417]	0.488 [0.225–1.058]
Green Access = Pubic Access as reference		

OR < 1 indicates a decrease in the likelihood of showing change in intensity; OR = Odds ratio; CI = Confident Interval; * *p* <0.050; ** *p* < 0.010; *** *p* < 0.001; ns = Not significant; Significant values of OR are in bold.
